# Scutellarin inhibition of the rosuvastatin uptake in rat hepatocytes and the competition for organic anion transporting polypeptide 1B1 in HEK293T cells

**DOI:** 10.1038/s41598-020-58303-0

**Published:** 2020-01-28

**Authors:** Jianming Liu, Yongmei Guo, Keqi Liu, Xiyong Ye, Fang Wang, Yanqi Xu, Chunhua Xia

**Affiliations:** 1 Clinical Pharmacology Institute, Nanchang University, Nanchang, Jiangxi 330031 China; 2 School of Pharmacy, JiangXi Medical College, Shangrao, Jiangxi 334000 China

**Keywords:** Hypertension, Drug development

## Abstract

In this report, we investigated the hepatocytic uptake of rosuvastatin when administered with scutellarin (a Chinese herbal medicine) in rats and the role of organic anion transporting polypeptide 1B1 (OATP1B1) plays in the uptake. Forty-eight rats were randomly divided into two groups according to the medicine administered: rosuvastatin alone and rosuvastatin in combination with a series concentration of scutellarin. Rosuvastatin concentrations in blood and liver were measured using the liquid chromatography–tandem mass spectrometry (LC-MS) method. The uptake was also measured in rat primary hepatocytes and OATP1B1 transfected human embryonic kidney 293 T (HEK293T) cells. The uptake was investigated under the optimal intake conditions. The rosuvastatin Cmax and AUC_0−∞_ in rat plasma increased 55% and 61%, respectively in the combination treatment group; and the liver scutellarin concentrations decreased 32%, 34%, and 33% at 1 h, 2 h, and 6 h, respectively. All scutellarin dosages (20, 50, and 100 μM) inhibited the uptake of rosuvastatin in rat primary hepatocytes (4.71%, 22.73%, and 45.89%). Scutellarin of 10 μM significantly inhibited the *in vitro* uptake of rosuvastatin in OATP1B1-HEK293T cells (P < 0.05), with an IC50 of 60.53 ± 5.74 μM. Scutellarin increases the plasma concentration of rosuvastatin and inhibits the uptake in rat primary hepatocytes and OATP1B1-HEK293T cells, suggesting a drug interaction between scutellarin and rosuvastatin and OATP1B1 as a potential mechanism.

## Introduction

The incidence of cardiovascular disease is on the rise worldwide. Hyperlipidemia, a contributor for cardiovascular disease, was characterized by abnormally elevated plasma levels of any or all lipids including total cholesterol, triglycerides, lipoprotein cholesterol, and apolipoprotein. Hyperlipidemia may cause deaths in patients with chronic congestive heart failure, cerebral infarction and senile dementia^1,2^. Particularly, low levels of high-density lipoprotein cholesterol and apolipoprotein are found to be risk factors for cardiovascular diseases. Currently, the first line medication for hyperlipidemia are statins, a class of HMG-GoA reductase inhibitors^3^. Statins significantly reduce blood lipid levels and have attracted great clinical and research interests because of their safety and efficacy. Statins of conventional doses reduce LDL-C by 30% and the risk of coronary events by approximately 30%^4^. Rosuvastatin, represents a “new generation” statin and reduces TC, LDL-C, and TG levels through inhibiting the enzyme 3-hydroxy-3-methylglutaryl coenzyme A reductase^5^.

Scutellarin (or sometimes breviscapine) is a type of phenolic chemical compound extracted from *Erigeron breviscapus*, which has been practiced to treat paralysis, rheumatism, gastritis, toothache, and fever. Scutellarin is also used to treat hyperlipidemia in China^6^. *In vivo*, scutellarin is highly distributed in hepatocytes, the same as the main target cells of HMG-GoA reductase statins^7^. Many Traditional Chinese Medicines (TCMs) active substances have significant effects on substrates of hepatic transporter and impact transport efficiency^8–10^. Transporters play a key role in drug pharmacokinetics and the competition for them is vital for drug uptake, distribution, and elimination^11,12^. The organic anion transporting polypeptide 1B1 (OATP1B1), has been related to rosuvastatin uptake by hepatocytes^13^. However, the mechanism of OATP1B1 involvement remains unclear, or whether scutellarin and rosuvastatin compete for OATP1B1. Therefore, in this study, we treated rats with rosuvastatin alone, or with a combination of rosuvastatin and a series concentration of scutellarin, to examine the plasma and hepatic levels of rosuvastatin in rats, as well as in OATP1B1 transfected HEK293T cells.

## Materials and Methods

### Animals

A total of 48 healthy male Sprague-Dawley rats weighing 230–270 g were obtained by the Experimental Animal Center of Nanchang University Medical College. Animals were housed in individual cages in an air-conditioned room at room temperature of 25 ± 1 °C with a 12 h light: 12 h dark period. The study was approved by the Ethical Committee of JiangXi Medical College, and all experiments were carried out with humane care according the Animal Care and Use Committee (ACUC) of Nanchang University Medical College. Rats were given free access to drinking water, after experiments, rats were euthanized by intraperitoneal injection of sodium pentobarbital with a dose of 200 mg/kg.

### Treatment and sampling

The rats were randomly divided into two groups with 24 rats in each group: the rosuvastatin group (rosuvastatin 10 mg/kg) and the combination treatment group (rosuvastatin 10 mg/kg + scutellarin 50 mg/kg), the concentrations used in the animal were transformed from clinical dose. The treatment was administered by gavage in the morning before feeding, and scutellarin was administered immediately following the rosuvastatin administration for the combination treatment group. In each group, 6 rats were maintained throughout the study and used for orbital vein blood sampling (0.15 ml) at 0 (before), 0.5, 1.0, 1.5, 2.0, 3.0, 5.0, 8.0, 12.0, 18.0, 24.0 h post treatment, to obtain the concentration-time profile data. Six of the remaining rats in each group were sacrificed at 1 h, 2 h, and 6 h post treatment (each time point) to sample the 0.5 ml of eyeball blood and the liver tissues. Blood was stored in heparinized tubes and the plasma was separated by centrifugation, then stored at −80 °C for further analysis.

### Reagents and equipment

Scutellarin was purchased from Kunming Double Star Technology Development Co., Ltd. (content: >98%, batch number: 20141016); standard rosuvastatin calcium purchased from Zhejiang Xindonggang Pharmaceutical Co., Ltd. (content: 99.5%, batch number: 20151220 RS); The internal standard atorvastatin was purchased from Medchemexpress Co., Ltd. (content: 99.9%, batch number: HY-17379); Mass spectrometric detection was performed on an API 4000+ triple quadrupole mass spectrometer AB SCIEX, Boston, USA); Chromatographic separation was performed with a Shimadzu LC20AD system (Kyoto, Japan) equipped with an autosampler, an LC‐20ADXR binary pump, a column oven and an online degasser. Western blotting was performed on Odyssey Infrared Imaging system (LI-COR Biosciences, USA); inverted optical microscope (CKX-41) (Olympus, Japan); carbon dioxide incubator (Heraeus Heracell 150, Germany); OATP1B1-HEK293T cell line was purchased from Shanghai Jikai Gene Co., Ltd.; methanol (ChromAR grade) and ethyl acetate (AR grade) was purchased from Merck company; DMEM and non-essential amino acids were purchased from Gibco, USA; fetal bovine serum was purchased from Hangzhou Sijiqing Co., Ltd.; hepatocyte growth factor was purchased from Chemicon; transferrin was purchased from Sigma USA; antibodies used for Western blot analysis were purchased from Proteintech Group or Abcam company; EDTA was purchased from Wuhan Zhongjian Company; PVDF film was purchased from Milipore, USA; all other reagents were of AR grade.

### Detection of rosuvastatin levels in plasma and liver tissues of rats

The liquid chromatography used Shimadzu Pack VP-ODS C18 column (150 mm × 2.0 mm, 5 μm) at the temperature of 40 °C using a mobile phase of deionized water supplemented with 0.1% (v/v) formic acid and 0.2 mM ammonium formate (solvent A) and acetonitrile (solvent B). The gradient was performed with a total flow at 0.3 mL/min as the following: 0–0.5 min 5%(B), 0.5-3.0 min 5-90% (B), 3.0-6.0 min 90%(B), 6.0–7.5 min 90–5%(B).

Preparation of the analytical plasma and liver tissues: Rat plasma of 100 μL in an EP tube was mixed with 20 μL of atorvastatin (internal standard, 10 μg/mL) and 30 μL of glacial acetic acid, vortexed for 30 s, then added 1 mL of ethyl acetate and vortexed for another 5 min. The sample was centrifuged at 2500 r/min for 10 min, and 1 mL of the supernatant was dried by termovap sample concentrator at 50 °C. The sample was then added with 100 μL of mobile phase to reconstitute, vortexed for 30 s and centrifuged at 12000 r/min for 2 min, and take 10 μL of the supernatant for analysis. Preparation of liver tissue samples: tissue was homogenized in a ratio of (tissue/0.9% sodium chloride injection = 1 g/2 mL) and cooled in an ice bath. After that, 0.5 mL of the homogenate was placed in a test tube, vortexed for 1 min, ultrasonically shaken for 1 min and centrifuged for another 5 min (10 000 r/min). Added 200 μL of the supernatant into a centrifuge tube, with 10 μL of 10 μg/mL of the internal standard atorvastatin solution and 30 μL of glacial acetic acid to the vortex. After shaking for 30 s, added 1 mL of ethyl acetate, vortexed for 6 min, centrifuged for 10 min, then pipetted 1 mL of the supernatant into a test tube and dried at 50 °C in a dry bath N2. Finally, 100 μL of mobile phase was added to reconstitute, vortexed for 30 s and then centrifuged for 2 min (12000 r/min), and 10 μL of the supernatant was used for analysis.

### Pharmacokinetic calculations

Pharmacokinetic analysis was performed using noncompartmental methods. Rosuvastatin plasma-concentration data were analyzed using DAS 2.1 Microsoft. The maximum rosuvastatin concentration (Cmax) and the corresponding peak time (Tmax) were determined from the respective observed plasma concentration–time data. Area under the concentration-time curve to the last sampling time (AUC_0-last_) was calculated using the linear trapezoid method. AUC0-∞ was calculated as the sum of AUC_0-last_/and C_0-last_/k, where C_0-last_ is the last sampled concentration and k is the elimination rate constant obtained from the regressed slope of ln-transformed terminal concentrations. Terminal half-life (t_1/2_) was determined from ln2/k (0.693/k). Clearance rate was calculated as intravenous dose divided by AUC0-∞^13^.

### Determination of rosuvastatin uptake in rat primary hepatocytes

The two-step perfusion method was applied to isolate rat primary hepatocytes for analysis of rosuvastatin uptake characteristics under combination treatment of scutellarin^14^. Briefly, hepatocytes suspension (1 × 10^6^/mL in DMEM) were seeded into 24-well culture plates. Treatments were as the following: rosuvastatin control group (20 μM rosuvastatin), and scutellarin combined treatment group (20 μM rosuvastatin combined with 20, 50, and 100 μM scutellarin). Then cells were subjected to incubation at 37 °C for 40 s in the incubator. The drug containing culture medium was then slowly removed and cells were washed 4 times with 0.5 mL of sterile water. Before LC-MS/MS, and samples were stored at −80 °C and received 3 times of freeze-dissolve treatment.

### Western blot for OATP1B1 expression in HEK293T

MOCK-HEK293T and OATP1B1-HEK293T cells at logarithmic proliferative phase were digested with trypsin to prepare cell suspension. SDS protein lysate was used to lyse the cells and cell proteins were extracted by centrifugation. The 2-quinolinic acid (BCA) Method was used to determine the total protein concentration. The proteins were transferred onto polyvinylidene fluoride membrane (PVDF) by gel electrophoresis using 10% sodium dodecylbenzene sulfonate polyacrylamide. Samples were blocked in 5% milk for 1 h at room temperature, and were then added with primary antibody of the rabbit-derived monoclonal antibody OATP1B1 and were incubated overnight at 4 °C. The membrane was briefly rinsed 3 times in TBST, 5 minutes each time. The secondary antibody, goat anti-rabbit antibody conjugated to horseradish peroxidase was added and incubated for 1 h at room temperature, and then rinsed the membrane 3 times with TBST, 10 minutes each time. ECL chemiluminescence developer was added for image acquisition by using the ODYSSEY imaging system.

### Determination of rosuvastatin uptake in OATP1B1-HEK293T cells

OATP1B1 plasmids were successfully constructed and then were transfected into HEK293T cells (Shanghai Jikai Gene Co., Ltd.). MOCK-HEK293T cells served as a blank control to compare the rosuvastatin uptake. The cells were resuscitated and seeded into 24-well culture plates. After the cells reached 80% confluence, the cells were washed three times with 1 mL of pre-warmed HBSS solution at 37 °C. Before the third wash, the well plate was incubated for 20 minutes at 37 °C^15^. After wash, cells were treated with 0.1 μM rosuvastatin (rosuvastatin group) or 0.1 μM rosuvastatin in combination with scutellarin 0, 1.0, 5.0, 10, 50, 100 μM respectively. Each well was incubated with 500 μL of the corresponding HBSS solution for 5 minutes, with a pH of 7.4. Then drug was removed quickly and rosuvastatin uptake was detected. The effects of scutellarin on the rosuvastatin uptake in OATP1B1 transfected HEK293T cells were assayed by LC-MS/MS. The method was validated by examining the selectivity, sensitivity, accuracy, precision, recovery, matrix effect and stability profiles of rosuvastatin^16^.

### Statistical analysis

Data are expressed as mean and standard deviation. Significance of comparisons between groups was tested using student’s *t* test or analysis of variance (ANOVA) test where appropriate. The SPSS12.0 software was used to analyze the pharmacokinetic parameters of the two groups. A P value < 0.05 was considered statistically significant for two-tailed tests.

## Results

### Method validation of mass spectrometric detection

**Figure 1:**
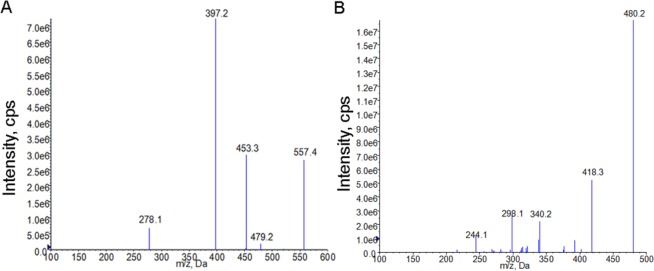
The ion transitions from precursor ion to product ion of (**A**) Resuvastatin, (**B**) Atorvastatin.

Mass spectrometric detection was performed on an API 4000+ triple quadrupole mass spectrometer (AB SCIEX, Boston, USA) equipped with an electrospray ionization (ESI) interface in negative ionization mode. Multiple-reaction monitoring (MRM) was used to monitor the analytes and the internal standard. The mass conditions were as follows: ion spray voltage, −4500 V; ion source gas 1 (N2), 60 Arb; ion source gas 2 (N2), 65 Arb; source gas temperature, 550 °C; curtain gas (N2), 30 Arb; collision gas (N2), 10 Pa; entrance potential, −10 V; cell exit potential, −12 V. The selected mass transitions were m/z 480.2 → 418.1 for RSV and m/z 557.6 → 397.1 for atorvastatin (IS), respectively (Fig. 1). The declustering potential (DP) was set at −80 V for rosuvastatin and atorvastatin. The collision energy (CE) was −21 eV for rosuvastatin and −39 eV for atorvastatin^17^. The selectivity of the method was evaluated by analyzing chromatograms of plasma samples from six different rats and cell samples to check for endogenous interference at the retention times of analytes and IS. The lower limit of quantification (LLOQ) was 2 ng/mL for plasma and liver, and was 1 ng/mL for cells. The dynamic range was 2–1200 ng/mL in plasma and liver and was 1–1000 ng/mL in cells respectively. Calibration curves were constructed by plotting the peak area ratios (analysts/internal standards) against the concentrations with weighted linear regression (weighing factor: 1/x, the correlation coefficients (R^2^) exceeding 0.99. Intra- and inter-day precision, accuracy, recovery, matrix effect and stability of rosuvastatin in rat plasma, liver tissue, primary hepatocytes and HEK293T cells are presented in Table 1.

**Table 1:** Intra- and inter-day precision, accuracy, recovery, matrix effect and stability of rosuvastatin in rat plasma, liver tissue and cells (n = 6).

Samples	Concentration (ng/mL)	Intra-day Precision		Inter-day Precision		Stability		Matrix effect %	Recovery %
		RSD%	RE%	RSD%	RE%	Stock solution	Short-term		
plasma	10	10.1	8.19	7.34	5.21	95.7	101	98.2	99.3
	100	7.75	3.64	3.07	0.100	96.4	98.0	101	96.7
	800	6.08	4.70	5.64	−2.77	98.8	96.3	99.0	95.6
	10	8.41	4.78	4.28	2.45	102	101	100	96.8
liver tissue	100	5.59	6.90	3.69	−2.76	98.6	93.6	99.3	103
	800	3.89	6.67	8.25	7.13	92.9	96.7	102	98.2
	5	6.56	3.84	1.87	2.38	96.3	98.3	101	102
cells	60	2.31	5.77	4.68	−0.810	99.1	96.5	99.5	99.3
	600	1.96	3.42	3.57	4.38	101	99.8	101	100

### Effects of scutellarin on rosuvastatin concentration in plasma and liver tissues in rats

Scutellarin significantly affected the pharmacokinetics of rosuvastatin in the rats. The peak concentration (Cmax) and the area under the curve (AUC) significantly increased (P < 0.05), while clearance rate significantly decreased (P < 0.05, Table 1). Compared with the rosuvastatin group, the combination treatment group had increased Cmax, AUC_0-last_, and AUC_0-∞_ of rosuvastatin about 55%, 60%, and 61%, respectively, while the clearance rate value was reduced by 40%. The differences in other pharmacokinetic parameters tmax and t_1/2_ were not significant (Table 2).

**Table 2:** Pharmacokinetic parameters of scutellarin, rosuvastatin, and rosuvastatin under combination treatment (n = 6, data presented as mean ± standard deviation).

Pharmacokinetic parameters	Rosuvastatin treatment (10 mg/kg)	Combination treatment: rosuvastatin + scutellarin	P value
Half life (t1/2, h)	4.47 ± 1.31	4.76 ± 1.53	0.731
Tmax(h)	2.05 ± 0.660	2.16 ± 0.582	0.765
Cmax(ng/mL)	545 ± 143	846 ± 202*	0.0153
Clearance rate(ml/h/·kg)	501 ± 69.0	307 ± 42.1**	0.000
AUC0-last (ng·/h·ml)	2.94 × 10^3^ ± 854	4.74 × 10^3^ ± 1.22 × 10^3^*	0.0161
AUC0-∞ (ng·/h·ml)	2.96 × 10^3^ ± 896	4.76 × 10^3^ ± 1.23 × 10^3^*	0.0223

Compared groups: rosuvastatin treatment vs. combination treatment (rosuvastatin + scutellarin). *Indicates difference compared with the rosuvastatin alone group (P < 0.05), **indicates great difference compared with the rosuvastatin alone group (P < 0.01).

**Figure 2:**
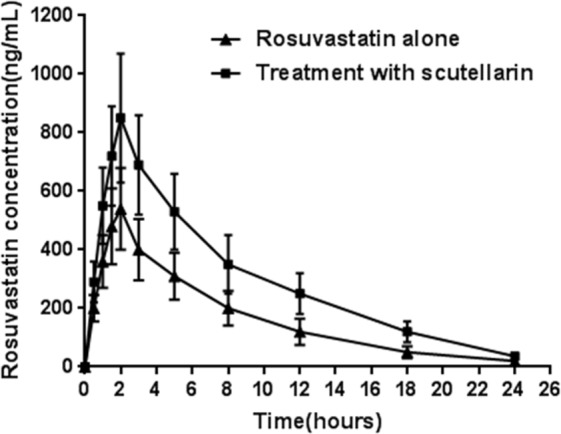
Mean plasma concentration–time profiles of rosuvastatin following an oral administration of rosuvastatin (10 mg/kg) to rats in the presence and the absence of scutellarin (50 mg/kg) (mean ± standard deviation, n = 6). Filled triangles rosuvastatin alone; filled quadrate treatment with 50 mg/kg of scutellarin.

The mean plasma concentration–time profiles of rosuvastatin in the presence and absence of scutellarin were characterized in rats and illustrated in Fig. 2. The result showed that scutellarin can signigicantly affect the features of rosuvastatin pharmacokinetics. It can increase the degree of absorption, but slow down the process of elimination of rosuvastatin.

**Figure 3:**
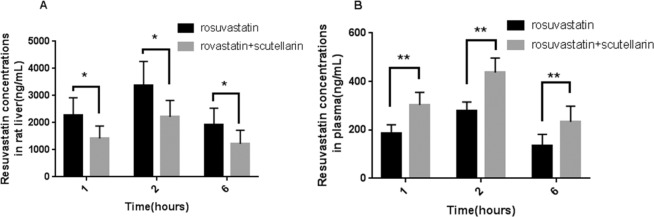
Rosuvastatin concentrations in liver tissues and plasma (n = 6, mean ± standard deviation). (**A**) Resuvastatin concentrations in rat liver tissues at different time points, (**B**) Resuvastatin concentrations in rat plasma at different time points. Compared groups: rosuvastatin treatment vs. combination treatment (rosuvastatin + scutellarin). *Indicates difference compared with the rosuvastatin alone group (P < 0.05), **indicates great difference compared with the rosuvastatin alone group (P < 0.01).

The rosuvastatin concentrations in liver tissues and plasma at different time points (1, 2, 6 h) in rats were compared rosuvastatin group and combination treatment (rosuvastatin + scutellarin) group. The results showed that the combination treatment significantly reduced the rosuvastatin concentration in the liver while increased the concentration in the plasma in rats (Fig. 3A,B).

### OATP1B1 expression in HEK293T by Western blotting

**Figure 4:**
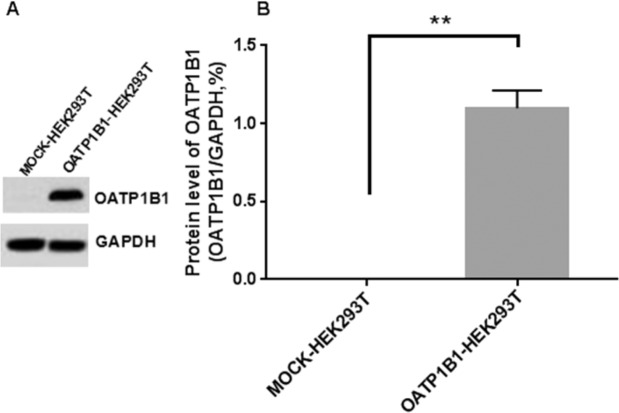
The expression level of OATP1B1 in MOCK/OATP1B1-HEK293T cells (**A**) photograph of western blot, (**B**) histogram of quantifying the grayscale value of western blot band (n = 3, mean ± standard deviation).Compared with MOCK-HEK293T, **indicates great difference compared with the MOCK-HEK293T group (P < 0.01).

The expression of OATP1B1 in each cell line was analyzed by western blotting. Membrane proteins of blank cells and transfected cell lines were extracted for detection, and sodium butyrate (2.5 mM) was induced by adding complete medium 24 h before cell collection. The results showed that the blank cells had almost no band around the 90KD marker (target protein 76KD), while the OATP1B1 transfected cell line showed a distinct band below marker 90KD, indicating that OATP1B1 was successfully expressed after transfection of HEK293T cells (Fig. 4).

### Effects of scutellarin on the rosuvastatin uptake in primary hepatocytes

The primary hepatocytes obtained from rats were strip-like or round-shaped, the cells were translucent with rich cytoplasm, and the boundary was clear. Small particles and the vacuoles were visible in the cytoplasm. The nuclei are medium-sized, round or oval, eccentrically placed, multi-nuclei or dual-nuclei, more common with a single nucleus. Cultured cells gradually became adhered, stretched, and the volume became significantly larger. Some of the cells expanded in a polygonal shape, and some cells were connected in an island shape.

**Figure 5:**
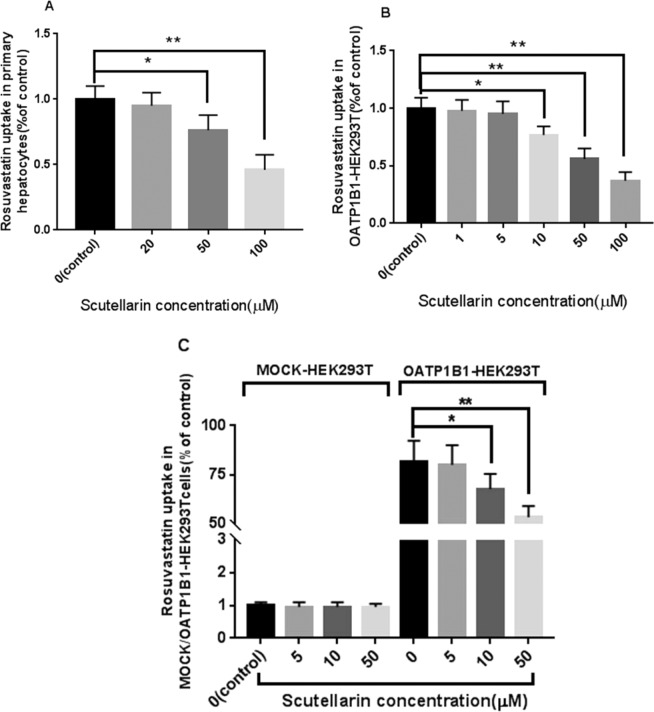
(**A**) Effects of scutellarin on rosuvastatin uptake in rat primary hepatocytes, (**B**) in OATP1B1-HEK293T cells and (**C**) The uptake of rosuvastatin in OATP1B1-HEK293T cells vs MOCK-HEK293T cell (n = 3, mean ± standard deviation). Compared groups: rosuvastatin treatment vs. combination treatment (rosuvastatin + scutellarin), *indicates difference compared with the rosuvastatin alone group statistically (P < 0.05), **indicates great difference compared with the rosuvastatin alone group (P < 0.01).

In rat primary hepatocytes, 20, 50 and 100 μM of scutellarin inhibited the uptake of rosuvastatin and reduced the uptake by 4.71%, 22.73%, and 45.89% respectively, as shown in Fig. 5(A). The 50 μM and 100 μM of scutellarin significantly inhibited the uptake of rosuvastatin by hepatocytes. However, the inhibition induced by 20 μM of scutellarin was not significant. With the increase of scutellarin concentration, the inhibitory effect on the uptake of rosuvastatin in hepatocytes was also enhanced.

### Effects of scutellarin on rosuvastatin uptake in OATP1B1-HEK293T cells

Under the microscope, OATP1B1-HEK293T cells were semi-adherent. Cells were flat under an inverted microscope. The single cells were triangular or irregular, and the cytoplasm was translucent with obvious epithelioid cell characteristics. After 96 h of culture, cell growth entered the plateau phase. The toxicity of 1–200 μM rosuvastatin and 1–150 μM scutellarin were tolerable for HEK293T cells, and cell viability remained above 80% under the microscope at the maximum concentrations. No significant cell floating was observed. In addition, considering the solubility of the drug, the subsequent concentration of rosuvastatin and scutellarin in OATP1B1-HEK293T cells was controlled between 1–150 μM.

The results showed that scutellarin reduced the uptake of rosuvastatin in OATP1B1-HEK293T cells, and the intake decreased along with the increase in dose of scutellarin. When the concentration of scutellarin was 10 μM, compared with the control group, the uptake was statistically reduced (p < 0.05). At the concentration of 5.0 μM and below, there was no statistical decrease in uptake (p > 0.05), see Fig. 5(B). The IC50 of the inhibition parameter was calculated to be 60.53 ± 5.74 μM in OATP1B1-HEK293T cells. The uptake of rosuvastatin by MOCK-HEK293T cells and OATP1B1-HEK293T cells was compared at four concentrations (0, 5,10, 50 μM; Fig. 5C). Rosuvastatin enters MOCK-HEK293T cells in very small amounts and is hardly affected by scutellarin. However, rosuvastatin uptake was significantly increased in the transfected OATP1B1-HEK293T cells. Scutellarin inhibited the uptake of rosuvastatin by HEK293T cells, and this inhibitory effect was positively correlated with the concentration of scutellarin in a certain range.

## Discussion

In China, statins are often prescribed to patients with a history or simultaneous use of TCMs which are believed to have the capacity to lower lipids^18^. However, the interaction between TCM and satins may cause pharmacokinetic changes and lead to severe/serious adverse events^19^. Perviously study suggests that intragastric administration of the TCM scutellarin affects the pharmacokinetics of rosuvastatin and rosuvastatin concentration in liver tissues in rats^20^. In our study, the combination treatment group increased the Cmax and AUC_0−∞_ of plasma rosuvastatin by about 55% and 61%, respectively; rosuvastatin concentration in the liver tissue decreased 32%, 34% and 33% at 1 h, 2 h, and 6 h, respectively. The results are consistent with literatures, indicating that scutellarin can inhibit the uptake of rosuvastatin by hepatocytes *in vivo* and reduce rosuvastatin concentration in liver tissues. Further studies in rat primary hepatocyte also found that scutellarin inhibited the uptake and transport of rosuvastatin *in vitro*.

OATP1B1 has an important influence on the *in vivo* pharmacokinetics of many drugs^21^. An increasing number of drugs are confirmed to be substrates of OATP1B1 mediated transport and are transported into the liver. Early studies have found that cyclosporine A can inhibit OATP1B1-mediated transport of cerivastatin, which increases the concentration of cerivastatin *in vivo* and changes the distribution of cerivastatin *in vivo*^22^. OATP mediates rosuvastatin transportation, cyclosporine A can competitively inhibit the uptake of rosuvastatin by the hepatocyte membrane OATP and thus increases drug plasma concentration^23^. Studies have also found that Salvianic acid A can inhibit OATP1B1 by competition, thus affecting the pharmacokinetic characteristics of rosuvastatin in rats^24^. In our study, the rat model and primary hepatocyte uptake experiments showed that the effect of scutellarin on the uptake of rosuvastatin in hepatocytes may be mediated by OATP1B1. We purchased OATP1B1-HEK293T cells, its stable overexpressing OATP1B1 was validated by western blot analysis; and the expression level was evaluated through observing the green fluorescent protein marker of OATP1B1-HEK293T plasmid vector by fluorescence microscopy. The transfection rate was determined to be greater than 80% and met the requirement of our design. HEK293T cells are currently one of the commonly used *in vitro* models for studying the relationship between drug transporters and substrates^25,26^. OATP1B1 transfected HEK-293T cells can express OATP1B1 and bind to transport ions. The results of our study showed scutellarin inhibited the uptake and transport of rosuvastatin in OATP1B1-HEK-293T cells.

Human OATP1B1 is expressed on the basement outer membrane of the hepatocyte membrane, and has a homologous transporter oatp1b2 in rat liver cells. There is a high degree of homology between human and rat liver transporters and studies in rat can be references for studies in human^27,28^. However, the alteration of OATPs expression did not completely parallel between human and rat primary hepatocytes, and there is no strict one-to-one relationship between human OATPs genes and rodent OATPs genes^29^. Rosuvastatin is mainly taken up by the liver, in which OATP1B1 plays an important role^30^. This study found that scutellarin had an effect on the pharmacokinetics of rosuvastatin in rats by intragastric administration. We also ruled out that this pharmacokinetic effect is due to the interference of scutellarin in the intestinal tract^31^, therefore, we consider that this inhibition of uptake occurs in the liver, which was supported by results from rat primary cell uptake experiments. Because hepatocyte only accounts for 10% of rosuvastatin metabolism^29^ and scutellarin has such a significant pharmacokinetic effect on rosuvastatin uptake, suggesting that this effect is more likely to occur on cell surface transporters. HEK293 cells do not express OATP1B1, and it is a model cell that is commonly used to investigate OATP1B1 transport^26^. In this study, we showed that OATP1B1 can mediate the inhibition of scutellarin on rosuvastatin uptake transfected HEK293T cells with high expression of OATP1B1. In addition, we also found that as for the admistration method, there is no pharmacokinetic difference between combining scutellarin 50 mg/kg with rosuvastatin at a single intragastric administration and pre-treatment with scutellarin 50 mg/kg once a day for 7 days before rosovastatin treatment^20^. Therefore, we suggest that the scutellarin inhibition on rosuvastatin uptake by liver should occur through competing for OATP1B1 in human hepatocytes.

In terms of drug dose selection, in the literature of pharmacokinetics and pharmacodynamics in rats, the doses of rosuravastatin were not consistent. Many scholars have given rosuvastatin to rats by intragastric administration of 5 mg/kg^32^. Xuan Zeng *et al*.^33^ used 50 mg/kg rosuvastatin for intragastric administration in rats for pharmacokinetic studies. For scutellarin, Hao X *et al*.^34^ used the dose of 10–60 mg/kg in rat pharmacokinetics. In human studies, JU Wenzheng *et al*.^35^ used oral scutellarin tablets at a dose of 120 mg to study the pharmacokinetics. Some scholars used intragastric administration of scutellarin at low, medium and high doses (25,50,100 mg/kg) for pharmacodynamic studies on liver fibrosis^36^. Combined with the results of our pilot experiment, this study selected 10 mg/kg rosuvastatin and 50 mg/kg scutellarin intragastrical doses for rats.

In summary, we investigated the effects of scutellarin on the uptake and transport of rosuvastatin in the liver through a rat model, primary hepatocytes and HEK-293T cell models. The results showed that scutellarin significantly inhibited the uptake of rosuvastatin through competing for transporter OATP1B1. The results of our study provide valuable reference for clinical use of the combination treatment of scutellarin and statins.
